# Early Achromobacter xylosoxidans Endocarditis After the Valvular Mitral Replacement Complicated by an Agranulocytosis on B-lactams: A Case Report

**DOI:** 10.7759/cureus.46045

**Published:** 2023-09-27

**Authors:** Aniss Channaoui, Michèle Dubus, Frédéric Mathieu, Séverine Noirhomme, Raphaël Fontaine

**Affiliations:** 1 Cardiac Surgery Department, Centre Hospitalier Régional Sambre et Meuse, Namur, BEL; 2 Cardiology Department, Centre Hospitalier Régional Sambre et Meuse, Namur, BEL; 3 Infectious Disease Department, Centre Hospitalier Régional Sambre et Meuse, Namur, BEL

**Keywords:** agranulocytosis, mitral valve surgery, adverse drug event, mitral endocarditis, achromobacter xylosoxidans

## Abstract

An immunocompetent 82-year-old woman developed endocarditis caused by an atypical organism called Achromobacter xylosoxidans, after a first valvular surgery. The intravenous antibiotic therapy with ceftazidime - 2 g every 8 hours during five weeks - a key part of the treatment, induced agranulocytosis as an adverse event. Cross-reactivity between antibiotics was suspected. Finally, the patient's cure was the result of a coordinated effort between medical and surgical professionals. Postoperative follow-up is six years.

## Introduction

*Achromobacter xylosoxidans* is a gram-negative bacterium that is typically found in aquatic environments [1]. In 2007, it has already been isolated in a contaminated chlorhexidine solution in a neonatal care unit [2]. We present a rare cause of endocarditis without continuous fever or elevated biological inflammatory syndrome three weeks after prosthetic mitral surgery. To our knowledge, only 21 cases of this nosocomial infection have been previously described [1]. The management of this pathogen is a medical and surgical challenge.

## Case presentation

**Figure 1:**
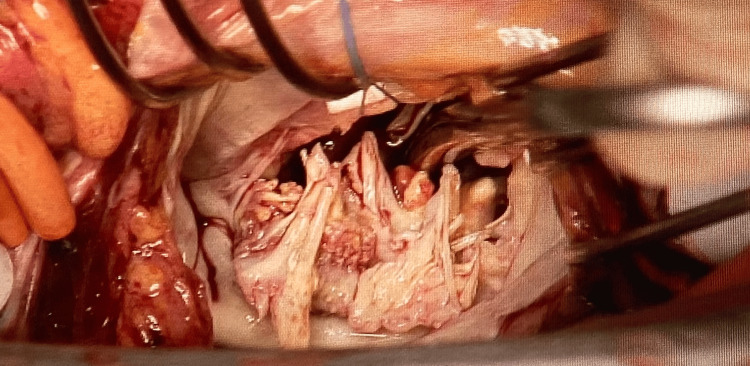
Perioperative view of the native mitral valve

An immunocompetent 82-year-old woman in excellent physical condition underwent a biological prosthetic mitral valve replacement for valvular stenosis (Figure 1).

Extensive annular decalcification was required, and atrioventricular continuity was reestablished using Carpentier's figure-of-8 technique without technical complication [3]. A 25-size Carpentier Edwards biological mitral valve (Edwards Lifesciences LLC, Irvine, CA) was then inserted. The immediate postoperative course was uneventful, and early anticoagulation was initiated. Three weeks later, the patient presented with septic symptoms (severe chills and short febrile episodes) without obvious clinical signs. The inflammatory markers were within normal limits. After a full bacteriological workup, the patient was treated for presumed deep urinary tract infection (new-onset urinary pain and dysuria) with intravenous (IV) vancomycin and ceftazidime in the context of suspected hospital-acquired infection, and recent valvular replacement. The patient remained afebrile from that point on. Two sets of blood cultures (BC) done before starting the antibiotics, identified *Achromobacter xylosoxidans*. Vancomycin was then discontinued according to the antibiogram (ceftazidime susceptibility).

**Figure 2:**
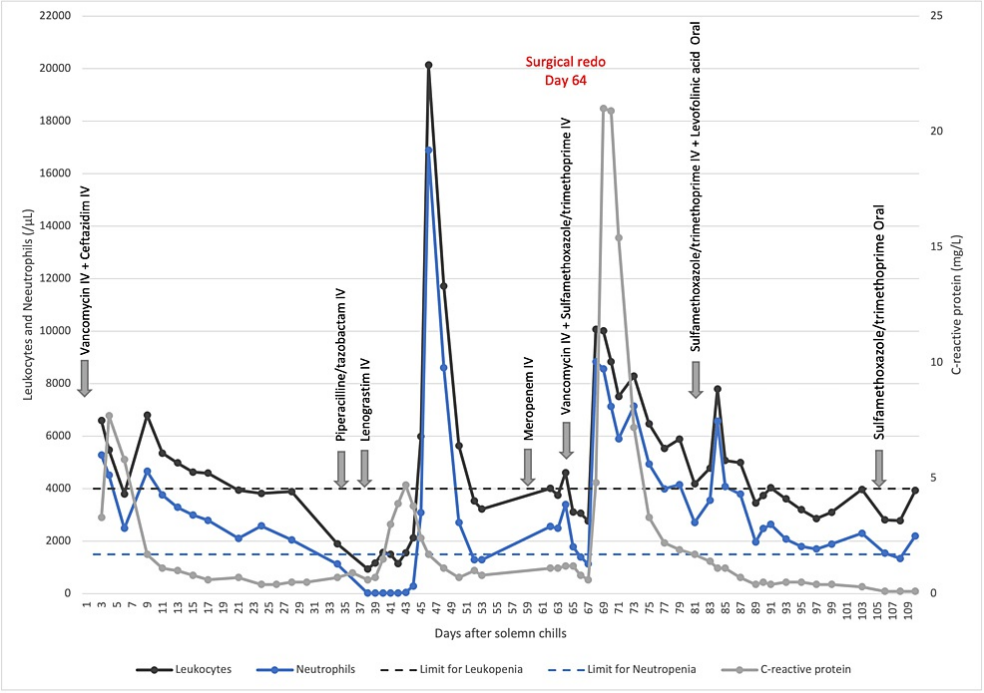
Patient’s biological course

A subsequent transesophageal echocardiography (TEE) revealed good left ventricular function, an image measuring 9 mm x 6 mm on the prosthetic valve, consistent with vegetation, and a thickening image lining the atrial wall extending into the left atrial appendage. They were not periprosthetic leaks or abscess images. After a multidisciplinary discussion, a conservative six-week course of IV ceftazidime was decided due to the patient's age and the high surgical risk of redo surgery. TEE was performed weekly to monitor vegetation and potential new-onset valvular insufficiency. The patient's clinical and biological evolution was favorable (Figure 2). Control BC remained negative.

On day 35 from initiation of empiric therapy, acute toxic leukopenia was diagnosed, and ceftazidime was switched to piperacillin/tazobactam. On day 38, piperacillin/tazobactam was stopped due to a lack of improvement in the white blood cell (WBC) count and because a cross-reactivity between piperacillin/tazobactam and ceftazidime was suspected. The same day, a granulocyte-colony-stimulating factor (G-CSF) was started for five days. Two days after piperacillin/tazobactam discontinuation, the WBC count increased. The last TEE control at patient discharge (day 50) showed a complete disappearance of the prosthetic cusp vegetation on the mitral valve leaflet and the absence of a periprosthetic leak or abscess. At this time, the patient was without antibiotics.

Nine days after discharge, the patient presented with transient aphasia, and brain magnetic resonance imaging demonstrated recent cortical ischemic lesions compatible with micro-embolic involvement. Laboratory results revealed a normal WBC count of 4010/μL, a C-reactive protein of 1.1 mg/dL, and an international normalized ratio (INR) of 4.53. TEE control demonstrated the presence of a mobile mass measuring > 10 mm on one of the prosthetic valve leaflets, consistent with new vegetation. They were not periprosthetic leaks or abscess images. BC came back positive for *Achromobacter xylosoxidans*. Meropenem was immediately administered according to the antibiogram, with careful hemogram monitoring.

Due to evidence of treatment failure with medication and resurgence of symptomatic prosthetic valve endocarditis (PVE), the cardiac surgical team performed a mitral valve replacement five days after readmission (day 64). The prosthetic valve was explanted, and extensive debridement was performed. The mitral annulus was reconstructed with an Edwards bovine pericardial patch. After annular reconstruction, different sizes of biologic mitral valves (25mm and 23mm - other sizes unavailable during surgery) were tested, but the matching was not optimal. An inverted 23 CE Magna Ease aortic valve (Edwards Lifesciences, Irvine, CA) was then implanted with acceptable transvalvular gradients. In view of the previous episodes of immune-allergic agranulocytosis due to beta-lactams (diagnosis confirmed by bone marrow biopsy), the patient was treated with high-dose IV sulfamethoxazole/trimethoprim. The explanted valve cultures confirmed *Achromobacter xylosoxidans* endocarditis (Table 1).

**Table 1:** Bacteriological analysis of explanted biological prosthetic mitral valve

Procurement	Vegetation	Minimum Inhibitory Concentration
Aerobic culture	positive	
Identification	Achromobacter xylosoxidans	
Temocillin	R	> 32,00
Piperacillin/tazobactam	S	< 4,00
Ceftazidime	S	4,00
Cefepime	R	16,00
Meropenem	S	< 0,25
Amikacin	R	> 64,00
Gentamicin	R	> 16,00
Ciprofloxacin	R	> 4,00
Cotrimoxazole	S	NA

After 19 days of IV sulfamethoxazole/trimethoprim (day 87), she presented a gradual decrease in all four WBC lines, requiring discontinuation of IV administration and a switch to a reduced oral dose of sulfamethoxazole/trimethoprim. This treatment was continued for a total of six weeks. After a multidisciplinary discussion, the patient was started on a daily, lifelong prophylactic dose of sulfamethoxazole/trimethoprim even if source control was achieved. The entire course of antibiotics administered to the patient is summarized in Table 2. The patient was discharged after 49 days, having fully recovered from her neurological accident.

**Table 2:** Timings and routes of drug administration

Timing	Prophylactic or therapeutic use/biological problem	Route	Drugs	Dose	Duration of administration
Day 0	Therapeutic	IV	Vancomycin	1g in 1hour, 1.5g daily	3 days
Day 0	Therapeutic	IV	Ceftazidime	2g every 8 hours	5 weeks
Day 35	Therapeutic	IV	Piperacillin/Tazobactam	4g every 6 hours	3 days
Day 38	Agranulocytosis	IV	Lenograstim	33.6 MUI daily	5 days
Day 59	Therapeutic	IV	Meropenem	1g every 8 hours	4 days
Day 64	Prophylactic	IV	Vancomycin	1g in 1hour, 1.5g daily	During cardiac surgery
Day 64	Therapeutic	IV	Sulfamethoxazole/ trimethoprim	2000/400mg every 12 hours	19 days
Day 82	Therapeutic	Oral	Sulfamethoxazole/ trimethoprim	1600/320mg every 12 hours	23 days
Day 82	Gradual fall in all four white blood cell lines	Oral	Levofolinic acid	7.5mg daily/7.5mg every 8 hours	13 days/10 days
From day 106	Prophylactic	Oral	Sulfamethoxazole/ trimethoprim	800/160mg daily	For life

The follow-up consisted of clinical and biological monitoring every six months, which showed no signs of inflammation or WBC variation. Transthoracic echocardiography was performed every six months initially and then annually, without any signs of endocarditis resurgence. The 88-year-old patient remains active and well without any signs of recurrence six years after surgery, be it biologically or at TEE control.

## Discussion

PVE is a rare but serious infection of a prosthetic heart valve. It is characterized by a high risk of morbidity and mortality, with an estimated in-hospital mortality rate of 20-40% [4]. The global incidence of PVE is 1-6% among patients with a prosthetic valve [5].

In this very specific situation of PVE, the medical and surgical team was challenged by the heterogeneity of the clinical presentation of endocarditis in terms of diagnosis and management [6]. The diagnosis of infectious endocarditis (IE) is more challenging to establish with a prosthetic valve than a native valve [7]. Fever is present in about 90% of cases, and the modified Duke criteria are typically used to make the diagnosis of IE [6,8]. However, when a prosthetic valve is in place, the sensitivity of these criteria is lower, and false-positive cases on echocardiography are possible (fibrosis, thrombi, or tumor) [6]. The 2023 European Society of Cardiology (ESC) guidelines recommend the eventual performance of a computed tomography (CT) scan or nuclear imaging in the event of doubt about the appearance of a new periprosthetic leak [5].

In our case, the main question of differential diagnosis between real vegetation or valvular thrombi on a bioprosthetic mitral valve arose. Considering the positive BC, the episode of severe chills on day 0, and the habit of our team to systematically anticoagulate bioprosthetic valves from the third postoperative day (for three months), the diagnosis of PVE was retained, as the valve culture confirmed. Six years ago, our center did not routinely perform complementary imaging in the face of suspected IE, as is currently recommended by the 2023 ESC guidelines. Since then, our diagnostic strategy has been revised in our center.

This patient has five of the most important prognostic factors for IE described in the scientific literature: advanced age, stroke, PVE, left-sided IE, and vegetation size greater than 10 mm [5,6]. A list of prognostic factors associated with poor outcomes in patient with IE has been published this year in the 2023 ESC guidelines: (1) Patient characteristics: Older age, PVE, haemodialysis, unsuitable for surgery, diabetes mellitus, high Charlson comorbidity index; (2) Clinical complications of IE: Heart failure, cerebral complications, septic shock, renal failure; (3) Microbiological features: *Staphylococcus aureus*, fungi, non-HACEK gram-negative bacilli, persistent bacteremia; (4) Echocardiographic findings: Periannular complications, left-sided infective endocarditis, vegetation size >10 mm, severe left-sided valve regurgitation, reduced left ventricular ejection fraction, pulmonary hypertension, prostheticvalve dysfunction, severe diastolic dysfunction echocardiographic signs of elevated left ventricular diastolic pressures [5].

Based on the prognostic factors for PVE, the theoretical prognosis of our patient was severe: older age, healthcare-associated infections, and early PVE [5]. Despite this, the patient is still alive and is the third oldest patient with *Achromobacter xylosoxidans* endocarditis reported to our knowledge after Rodrigues et al. (86 years old, aortic valve, alive on follow-up) and Tokuyasu et al. (86 years old, prosthetic aortic valve, dead on follow-up) but the oldest for mitral endocarditis, out of the only 21 previously published cases [2,9-13].

With this case, we have begun to reflect on the management of PVE in our center. The ideal management is not yet fully agreed upon by the different scientific societies, but valve replacement without delay is becoming increasingly accepted within the six months of initial valvular surgery (Class of recommendation I; Level of evidence C) [5]. Initially, we opted for a pure antibiotic treatment plan given the patient's postoperative clinical condition, the high morbidity and mortality associated with a new mitral valve replacement, and especially the initial refusal of the patient's family for further surgery. The patient initially had a good clinical course. However, the patient was readmitted to the hospital for a stroke. This time, a valve replacement was performed without delay due to evidence of uncontrolled infection under antibiotic therapy alone and a persistent high embolic risk (mobile vegetation > 10mm on the prosthetic valve), as recommended by the 2023 ESC guidelines.

Therefore, we remarked that it is important to rapidly use noninvasive imaging in addition to TEE as soon as PVE is suspected. Additionally, immediate reintervention with complete debridement and valve replacement, along with aggressive antibiotic therapy, is necessary in cases of uncontrolled infection. The atypical and rare nature of the pathogen involved in this case added an additional challenge to our therapeutic management.

## Conclusions

In conclusion, management guidelines are non-existent due to the unusual nature and high intrinsic resistance of *Achromobacter xylosoxidans*, which is often associated with immunosuppression. In our case, the broad-spectrum antibiotic therapy eventually induced an adverse event (iatrogenic immunosuppression) by generating a medullary aplasia that could have affected the patient's prognosis. Finally, as the very limited available literature suggests, the combination of long-term antibiotic treatment and surgical valve replacement is the most likely course of action to cure the patient.
